# The Rise of Telemedicine in Orthopaedic Trauma Follow-Up Care and Its Long-Term Outcomes: A Narrative Review

**DOI:** 10.7759/cureus.97896

**Published:** 2025-11-26

**Authors:** Ahmed Mohamed, Alaa Elasad, Usman Fuad, Ioannis P Pengas, Mohamed Abdelazim

**Affiliations:** 1 Trauma and Orthopaedics, Royal Cornwall Hospital, Truro, GBR; 2 General Practice, Zagazig University, Zagazig, EGY; 3 Internal Medicine, Royal Cornwall Hospital, Truro, GBR

**Keywords:** orthopaedic trauma, remote patient monitoring, telemedicine, virtual consultation, virtual fracture clinic

## Abstract

Telemedicine has rapidly transformed healthcare delivery, especially following the COVID-19 pandemic, which accelerated its use across nearly all medical specialties. Virtual consultations have become an integral part of patient follow-up and management in orthopedic trauma care. This review explores the evolution and current applications of telemedicine in orthopaedic trauma, highlighting its implementation and patient and clinician responses. We examine evidence comparing virtual and in-person care in terms of clinical outcomes, cost-effectiveness, and accessibility. This review also discusses the common barriers to adoption, practical solutions, and strategies that promote successful integration. Finally, we consider the long-term sustainability of telemedicine platforms and outline future directions for virtual orthopaedic trauma services. Together, these insights aim to guide ongoing efforts to optimize patient care delivery in the digital era.

## Introduction and background

Telemedicine represents the remote delivery of healthcare services using communication technology to connect providers and patients [1]. Its medical application dates back several decades; however, its widespread adoption in orthopedics remained limited until recently [2]. The COVID-19 pandemic changed everything as healthcare systems faced unprecedented challenges, where social distancing became essential, traditional clinic visits posed infection risks, and hospitals needed alternatives to face-to-face care [3,4]. Telemedicine offered a solution with the relaxation of regulatory restrictions during the pandemic, accelerating its adoption in orthopedic practices. Before the pandemic, orthopaedic practices in the United Kingdom had not widely utilized telemedicine, but the crisis forced rapid implementation, where what took years to develop happened in weeks [5].

Orthopaedic trauma poses unique challenges in virtual care. Physical examination depends mainly on clinical and radiological assessments. Clinical evaluation includes pain and range of motion assessment, which requires an in-person examination. These factors made surgeons hesitant to use telemedicine. Despite these concerns, trauma follow-up was found to be suitable for virtual platforms. Many postoperative visits involve wound checks, patients need reassurance about healing progress, questions about activity restrictions arise frequently, and pain management requires ongoing adjustments. These needs can often be addressed remotely [6,7].

This review examines telemedicine in orthopaedic trauma follow-up care, focusing on long-term outcomes and sustainability. The goal is to provide evidence for continued implementation, where understanding benefits and limitations guides future practice.

## Review

Methods

Search Strategy

A comprehensive literature search was conducted to identify relevant studies on telemedicine in orthopaedic trauma follow-up care. PubMed, Web of Science, and Google Scholar databases were searched from January 2015 to October 2025. The search strategy included combinations of keywords such as "telemedicine", "telehealth", "virtual consultation", "orthopedic", "orthopaedic", "trauma", "fracture", "follow-up", and "outcomes". Additional articles were identified through manual screening of the reference lists of relevant systematic reviews and meta-analyses.

Selection Criteria

We included studies that met the following criteria: (i) published in English, (ii) focused on telemedicine applications in orthopaedic trauma or fracture follow-up care, (iii) reported outcomes related to patient satisfaction, provider satisfaction, clinical outcomes, cost-effectiveness, or implementation experiences, and (iv) included randomized controlled trials, cohort studies, systematic reviews, or descriptive studies with large sample sizes. We excluded case reports, editorials without original data, and studies focusing solely on preoperative consultations or rehabilitation without trauma-specific content.

Data Extraction and Synthesis

Data were extracted from the included studies, including study design, sample size, patient population, telemedicine intervention type, comparison groups, outcome measures, and key findings. Given the heterogeneity of the study designs and outcome measures, we performed a narrative synthesis of the evidence. Studies were organized thematically around key topics, including historical evolution, current applications, patient and provider satisfaction, clinical outcomes, cost-effectiveness, barriers, and future directions.

Characteristics of Included Studies

Table 1 summarizes the characteristics of some key included studies that form the basis of this narrative review.

**Table 1:** Summary of Key Studies Included in the Narrative Review RCT: randomized controlled trial; ADLs: activities of daily living; N/A: not applicable

Study	Year	Study Design	Sample Size	Population	Intervention	Key Findings
Sathiyakumar et al. [8]	2015	RCT (pilot)	24	Orthopedic trauma patients	Video telemedicine vs in-person follow-up	Similar patient satisfaction; significantly less time and distance traveled
Buvik et al. [9]	2019	RCT	389	General orthopedic patients	Remote video consultation vs in-person	No difference in patient-reported outcomes; high satisfaction
Buvik et al. [10]	2019	RCT (cost analysis)	389	General orthopedic patients	Telemedicine vs standard care	Cost-effective; savings increased with distance
Haider et al. [2]	2020	Systematic review	21 studies	Orthopaedic populations	Various telemedicine modalities	High satisfaction; cost-effective; safe; valid examination
Rizzi et al. [5]	2020	Cross-sectional study	612 encounters	Adult orthopaedic patients	Phone and video telemedicine	93% patients willing to use again; 78.4% visits successfully replaced in-person
Makhni et al. [11]	2020	Review	N/A	Orthopaedic surgery	Telemedicine overview	Barriers include physical exam limitations, costs, and regulatory issues
Gilbert et al. [12]	2021	Survey study	381	Orthopaedic patients	Patient barriers assessment	18.5% reported barriers; age >61, non-English, inexperience associated
Hurley et al. [13]	2021	Survey study	268	Orthopaedic surgeons	Surgeon perspectives	Higher satisfaction for follow-up than new patients; most plan to continue
Chaudhry et al. [14]	2021	Systematic review and meta-analysis	Multiple studies	Orthopaedic patients	Telemedicine satisfaction	High patient and surgeon satisfaction during COVID-19
Fahey et al. [15]	2021	Systematic review	41 studies	Orthopaedic populations	Telemedicine outcomes	2 showed noninferiority, 9 no difference, 4 superior outcomes
Muschol et al. [16]	2023	RCT	60	Knee/shoulder conditions	Video vs in-person follow-up	Slightly higher satisfaction with video; no significant difference
Hofmann et al. [17]	2023	Systematic review	Multiple studies	Orthopaedic/trauma surgery	Telemedicine during COVID	Efficacy varies by application; high adoption during COVID pandemic
Talaski et al. [18]	2024	Systematic review	12 studies	Foot and ankle orthopaedics	Telemedicine usage	Equivalent outcomes; patients preferred in-person for future
Pliannuom et al. [19]	2024	Meta-analysis	16 studies	Hip fracture patients	Telerehabilitation	Improvements in functional outcomes and ADLs

Historical context and evolution

Telemedicine in orthopaedics began modestly, with early applications focused on remote consultations between physicians. Lambercht et al. analyzed 410 orthopaedic teleconsultations performed over a two-year period and found that fracture evaluation and treatment represented 43% of consultations [20]. These pioneering efforts demonstrated feasibility, while technological improvements drove expansion as video quality improved. Internet connectivity became more reliable, and mobile devices gained widespread adoption [21]. Prior to the COVID-19 pandemic, telemedicine in orthopaedics and trauma surgery had mostly developed for joint arthroplasty, fracture management, and general pre- and postoperative care; however, applications remained limited to specific cases, and many surgeons remained skeptical [17].

The pandemic forced a paradigm shift with rapid adoption, and patients reported high satisfaction levels, with 92.9% willing to complete another telemedicine encounter [5]. What began as an emergency response became standard practice, where surgeons who had never used telemedicine became proficient quickly. Virtual fracture clinics (VFCs) have emerged as important innovations, with the Glasgow Fracture Pathway pioneering this approach in 2011 [22,23]. This alternative pathway aimed to reduce traditional fracture clinic visits, waiting times, and overall costs for managing simple stable fractures, where the model proved successful and spread globally. Telerehabilitation has also gained traction as physical therapy delivered remotely, with studies in hip fracture populations demonstrating improvements in functional independence, activities of daily living, and quality of life [24].

Current applications in trauma and orthopaedic care

Telemedicine has become an integral component of trauma and orthopaedic practice, facilitating both conservative and operative management. For conservatively managed injuries, such as stable fractures, sprains, or soft-tissue trauma, virtual consultations often provide equivalent value to face-to-face visits [17]. During virtual consultations, clinicians can review patients' radiographs, confirm that injuries are isolated, closed, and neurovascularly intact, and suggest the treatment of choice. Patients can follow up remotely without repeated hospital visits until complete healing and functional recovery [25]. Pain management and medication review can also be conducted virtually with real-time symptom reporting and dose adjustment, improving safety and patient satisfaction [26].

Telemedicine is now used throughout the perioperative pathway in operative cases. During the preoperative phase, virtual consultations allow surgeons to obtain a detailed history, review investigations, and discuss the consent process, including surgical indications, operative risks, and expectations using visual aids [27]. Preoperative tele-assessment has been shown to improve efficiency, streamline surgical planning, and minimize unnecessary hospital visits [28]. Postoperatively, telemedicine plays a crucial role in the follow-up. The most common use is virtual wound assessment, allowing patients to show their incisions via secure video platforms, enabling early identification of infection or delayed healing [29]. Virtual fracture and implant follow-up have become more common with remote radiograph submissions, allowing surgeons to evaluate alignment, union, and implant integrity [30]. Additionally, telerehabilitation using video sessions, mobile applications, virtual reality, and wearable sensors facilitates guided physiotherapy and progress tracking at home. This approach reduces clinic burden, costs, travel, and time lost from work while enhancing accessibility and supporting continuity of care, particularly in rural or resource-limited settings [19].

Implementation models and platforms

Various models exist for telemedicine delivery, with phone-only consultations representing the simplest approach, allowing rapid patient communication and triage but lacking visual assessment. Video consultations offer more information through platforms like Zoom (Zoom Communications, Inc., San Jose, California, United States), Microsoft Teams (Microsoft Corporation, Redmond, Washington, United States), and proprietary systems that were developed by healthcare systems during the pandemic, where video allows partial physical examination, as surgeons can observe gait and movement, and wound appearance can be assessed [31]. Asynchronous telemedicine provides an alternative model in which patients upload clinical information, radiographs, photographs, or videos at their convenience. Surgeons later review the submissions and provide tailored feedback or treatment recommendations, where this store-and-forward model offers flexibility, efficiency, and reduced time constraints for both patients and clinicians [32].

Hybrid models combine in-person and virtual consultations, often adopted in VFCs. In these systems, initial assessments are performed remotely, and studies have reported that up to 75% of patients can be managed conservatively without further face-to-face review. However, successful implementation requires experienced clinicians capable of appropriately selecting patients for remote versus in-person assessments to ensure safety and quality of care [33,34]. Advancements in mobile health applications have further expanded telemedicine capabilities, facilitating appointment scheduling, secure communication, and medical record storage while enabling patients to log symptoms, pain levels, and rehabilitation progress [35]. The integration of health records ensures seamless data flow and improves clinical efficiency. Additionally, remote monitoring devices, including wearable sensors and smart devices, allow continuous tracking of physical activity, range of motion, and physiological parameters, where the automated transmission of these data to clinicians supports objective assessment of recovery and early detection of complications [36].

Patient satisfaction and experience

Patient satisfaction with telemedicine is remarkably high, with multiple studies reporting positive experiences. Studies have revealed high patient satisfaction due to convenience, reduced waiting time, and decreased travel requirements [37]. A randomized controlled trial found that patients using telemedicine experienced similar satisfaction to in-person visits, but with significant savings in time and effort [8]. Convenience ranks as a top satisfaction factor, where patients appreciate avoiding clinic visits, value flexibility in scheduling, and home-based consultations reduce stress, particularly for trauma patients with mobility limitations [38]. Rural patients benefit substantially from telemedicine, as studies of trauma centers have found that patients admitted from long distances face significant follow-up care challenges, which telemedicine has helped address, where long travel distances for follow-up represent a burden that has been eliminated [39].

Elderly patients can adapt successfully to telemedicine despite initial concerns about technology use. Studies have found that although barriers included age over 61 years, the majority of elderly patients still reported satisfaction. With appropriate support, older adults engage effectively, with family members often assisting with technology [40,41]. Technological confidence affects satisfaction. Patients who are comfortable with video calls adapt easily. Among patients willing to try telemedicine before COVID-19, the satisfaction rate reached 97.1% [12]. Those initially resistant to telemedicine often become satisfied after experiencing it, where education about the benefits helps acceptance. However, some patients still prefer in-person visits despite satisfaction, as studies show that many prefer in-person visits for future appointments [18]. Familiarity with traditional care persists, and physical presence provides reassurance for some, where this preference varies by individual.

Provider satisfaction and perspectives

Surgeon satisfaction with telemedicine varies but is generally positive, with physicians reporting high satisfaction at 78.4%, supporting the notion that telemedicine can replace in-person appointments [5]. Many initially skeptical surgeons became advocates, with most physicians having no prior telemedicine experience but becoming comfortable after completing just one to four training sessions [5]. Surgeons appreciate telemedicine benefits as they increase productivity, where scheduling appointments becomes easier and more efficient; telemedicine consultations averaged shorter duration compared to in-person visits, more patients can be seen, and travel time between clinics is eliminated [42].

However, concerns about physical examination persist, as physicians have neutral attitudes toward telemedicine, with concerns about the lack of physical examination capabilities [18]. Physicians raise concerns about missing subtle findings, as the examination depends mainly on the patient's description of symptoms, which can sometimes be subjective. Visit type affects surgeon satisfaction, with surgeons reporting higher satisfaction with telemedicine for routine follow-up and postoperative patients than for new patients [13]. Surgeons find it more comfortable to see patients initially with an in-person consultation to have a baseline of knowledge about the patient's symptoms and build an idea about complications that might happen. Technical issues frustrate providers; the key barrier identified for telemedicine was technical difficulties, where poor internet connections disrupt consultations and audio problems impair communication, and platform reliability is essential [43]. Medicolegal concerns also worry some surgeons as documentation requirements need clarification, and liability for remote diagnoses is uncertain. Multiple legal and ethical concerns persist, including the liability of clinicians performing remote examinations and the responsibility for privacy and confidentiality, where clear guidelines would help [44].

Clinical outcomes and safety

Evidence to date suggests that telemedicine delivers clinical outcomes comparable to, and in some cases, superior to traditional face-to-face orthopaedic care. Across 15 comparative studies, two demonstrated non-inferiority, nine reported no statistically significant difference, and four found telemedicine to be superior in overall clinical performance [15]. This growing body of evidence supports the clinical safety and efficacy of virtual care models in trauma and orthopaedics. Safety profiles appear equivalent between telemedicine and in-person care, with no increase has been observed in self-reported adverse events, postoperative complications, or delayed diagnoses when care is delivered virtually [2,9]. In fracture management, outcomes remain satisfactory, fracture healing progresses normally, malunion and non-union rates are not elevated, and radiographic monitoring of alignment and callus formation remains reliable using digital imaging uploads [8].

Functional outcomes following operative treatment, particularly after hip and knee arthroplasty, are also encouraging. Multiple studies report no significant differences in postoperative disability indices, range of motion, muscle strength, or patient-reported quality-of-life measures between telemedicine and traditional follow-up groups [9]. In hip fracture populations, telerehabilitation has been associated with improved mobility scores, functional independence, and adherence to home-based exercises, facilitated by greater convenience and reduced travel burden [19]. Follow-up adherence is notably better with telemedicine, with non-attendance rates as low as 2.7% in some series [5]. Enhanced accessibility promotes consistent monitoring, timely review of concerns, and early intervention when needed. Importantly, unplanned healthcare utilization, including emergency visits and unscheduled appointments, remained comparable between the virtual and in-person groups, indicating that telemedicine does not compromise patient safety or delay the recognition of complications [2,9].

Quality-of-life outcomes mirror these findings, where standardized health-related assessments demonstrate equivalent improvement at three months post-intervention, with high patient satisfaction and psychological reassurance attributed to easier communication and continuity of care [16]. Encouraging results have also been reported in special populations, including pediatric fracture management, where remote monitoring of surgically treated supracondylar humerus fractures achieved excellent results with high satisfaction [45].

Cost-effectiveness and resource utilization

Economic evaluations have demonstrated that telemedicine is a cost-effective alternative to conventional orthopaedic care. In nine comparative studies, telemedicine interventions were associated with significant cost reductions for both patients and healthcare systems [15]. These savings arise from several interrelated factors, where at the patient level, telemedicine eliminates travel expenses, parking fees, and time lost from work [2]. For individuals living in rural or remote areas, the reduction in travel distance and appointment-related disruptions translates into meaningful financial and time savings [10]. Additionally, indirect costs such as childcare, caregiver burden, and lost productivity are minimized, contributing to improved accessibility and equity of care [46].

From a healthcare system perspective, telemedicine optimizes resource utilization, where economic analyses have shown measurable reductions in overhead expenses and staff workload, as well as more efficient use of clinic space [16]. Virtual follow-up enables hospitals to reallocate outpatient capacity toward new or complex cases while maintaining continuity of care for stable patients. In trauma care, VFCs represent a particularly cost-effective model that maintains a thorough review of all trauma patients and enables the filtering of patients and setting plans remotely without compromising outcomes and reducing unnecessary radiographs and follow-up visits [34]. This model not only decreases direct care costs but also enhances system efficiency by reducing emergency department congestion and waiting times.

Although the initial expenses for setting up, such as buying hardware, obtaining software licenses, and training staff, can be significant, these costs are generally balanced out as telemedicine initiatives develop over time [11]. Efficiency gains, reduced patient no-shows, and sustained patient engagement contribute to long-term cost recovery and revenue stability, and as telehealth volume increases, economies of scale reinforce financial sustainability [47]. Reimbursement policies play an important role in maintaining sustainable telemedicine. Recent changes have allowed many healthcare systems to pay doctors equally for telemedicine and in-person visits, making virtual care more financially viable [48]. Continuing these policies will help ensure that telemedicine remains a cost-effective and accessible option in trauma and orthopedic practice.

Barriers to implementation and adoption

Despite its many advantages, several challenges continue to limit the widespread use of telemedicine in trauma and orthopedic practice. These barriers can be grouped into technical, social, professional, and regulatory categories. Technical barriers remain among the most common, and many patients lack access to suitable devices, reliable Internet connections, or private spaces for virtual consultations [49]. This is especially problematic in rural and low-resource areas, where poor connectivity and limited infrastructure restrict access to virtual care, creating disparities in healthcare delivery and limiting the potential reach of telemedicine [39].

Digital literacy is a major obstacle. Older adults or those with lower educational backgrounds may feel uncomfortable using video platforms or online portals without support [50]. A lack of confidence or prior experience can lead to frustration and reluctance to engage in telemedicine; therefore, providing education, technical support, and simplified user interfaces can help overcome these barriers [50]. Language and socioeconomic factors also affect access; for example, one study found that over 96% of telemedicine visits were conducted in English, despite only 61% of the local population reporting English as their first language [51]. Without translation services and culturally sensitive approaches, non-English-speaking or minority patients risk being excluded, and similarly, lower-income groups are underrepresented among telemedicine users, contradicting the goal of equitable access.

At the institutional level, infrastructure and resource limitations remain problematic, as not all healthcare systems have integrated telemedicine platforms or sufficient IT support [52]. Effective implementation requires investment in technology, staff training, and clear operational workflows to ensure quality and consistency of care delivery [1]. Regulatory and legal challenges further complicate adoption, as licensing requirements, reimbursement policies, and privacy regulations differ between regions and can make telemedicine implementation complex [20]. Interstate or international consultations face additional legal and credentialing hurdles, while concerns about data security and medicolegal liability persist [44].

Finally, professional and clinical barriers play an important role, with some surgeons remaining hesitant to adopt telemedicine due to unfamiliarity with the technology, changes in workflow, or skepticism about clinical reliability. Orthopaedics, in particular, depends heavily on physical examination, including palpation, stability testing, and joint manipulation, which cannot be fully replicated virtually [15,53]. Acknowledging these limitations and developing hybrid models that combine virtual and in-person assessments may help build clinician confidence and improve patient safety.

Solutions and best practices

Overcoming barriers to telemedicine adoption in trauma and orthopaedic practice requires a structured multilevel approach involving patients, clinicians, healthcare systems, and policymakers. Education is the foundation of successful telemedicine use. Patients should be informed of the benefits and limitations of virtual care through clear communication and shared decision-making [54]. Likewise, healthcare providers need structured training on telemedicine etiquette, remote assessment techniques, and virtual examination skills to build confidence, ensure safety, and maintain clinical standards [52].

Patient selection is crucial for ensuring safety and efficiency. Clear criteria should guide which patients can be reviewed virtually, such as stable fractures, wound checks, or postoperative follow-ups, while complex or uncertain cases should be prioritized for in-person evaluation [2]. Screening tools based on known barriers, such as limited technology access or poor digital literacy, can identify patients who may need additional support before virtual visits. Reliable technical assistance greatly improved the success of telemedicine. Patients should have access to help lines, video tutorials, and test calls before appointments [55]. Healthcare organizations should invest in user-friendly, secure platforms that integrate electronic medical records and comply with data-protection regulations. Involving caregivers or family members can further improve usability, particularly for elderly patients [56].

Standardized workflows enhance consistency and efficiency, using structured protocols, documentation templates, and checklists to ensure that virtual consultations meet clinical and medicolegal standards. Efficient scheduling systems and adequate logistical planning minimize disruptions and improve the overall service quality. Combining virtual and face-to-face consultations often provides the best balance, with surgeons reporting higher satisfaction when telemedicine is used for routine follow-up or postoperative reviews, while new or complex patients are seen in person [52]. Hybrid models allow for flexibility, reduce travel and waiting times, and maintain clinical safety.

Supportive policies are essential for sustained adoption, and reimbursement parity between virtual and in-person consultations must be maintained to ensure financial viability [48]. Simplifying licensure and regulatory processes, particularly for interstate or cross-regional care, will facilitate wider implementation. Therefore, ensuring equitable access remains a priority. Vulnerable and underserved populations, such as those with limited digital access, language barriers, or lower income, require targeted outreach and support [57]. Providing affordable technology, translation services, and culturally sensitive care can help prevent widening health disparities.

Long-term sustainability and future directions

Ensuring the long-term sustainability of telemedicine in trauma and orthopaedic practice requires continued efforts across clinical, technological, regulatory, and educational domains. While the COVID-19 pandemic accelerated adoption, telemedicine use declined once restrictions eased, particularly in procedure-based specialties such as orthopaedics [58]. Maintaining progress requires deliberate action, ongoing evaluation, and a clear demonstration of value to both clinicians and patients. Sustained telemedicine depends heavily on supportive policies. Many temporary measures introduced during the pandemic, such as relaxed licensure rules and expanded reimbursement, need to become permanent [11]. Ongoing advocacy by professional bodies and policymakers is essential for securing long-term financial and legal stability. Clearer medicolegal frameworks will also help clinicians practice confidently by defining standards of care, liability, and patient safety expectations [48].

Future advances in technology will further enhance telemedicine capabilities. Artificial intelligence can assist in diagnostic interpretation, automate image analysis, and personalize follow-up schedules [59]. Virtual and augmented reality tools show promise in improving patient education and rehabilitation, while wearable devices and remote monitoring systems generate continuous data on movement, healing, and function [60]. The integration of these technologies into a unified platform could create comprehensive data-driven virtual care ecosystems. Despite the growing literature, most existing studies are short-term and observational, and high-quality randomized controlled trials are needed to evaluate long-term outcomes, cost-effectiveness, and patient satisfaction [2,17]. Understanding how telemedicine affects recovery trajectories, complication rates, and healthcare utilization over years, not just months, will guide evidence-based implementation.

Sustainability also depends on embedding telemedicine in medical education. Training programs for residents and consultants should include virtual examination techniques, communication skills, and remote clinical reasoning [61]. Continuing professional development should reinforce best practices and ensure that providers maintain their confidence and competence as technology evolves. The establishment of quality metrics will be critical to maintaining standards, where routine measurement of outcomes, patient satisfaction, and process efficiency will support benchmarking and continuous improvement. Standardized protocols and evidence-based pathways, such as those already developed for VFCs managing injuries like fifth metatarsal or radial head fractures, will help define safe and effective telemedicine use [62].

Globally, telemedicine offers a way to bridge access gaps, particularly in resource-limited settings, where international collaboration could promote knowledge exchange, uniform standards, and global equity in orthopaedic care delivery [63]. Ethical and medicolegal frameworks will continue to evolve, ensuring that patient confidentiality, autonomy, and informed consent are upheld. Finally, the future of telemedicine must remain patient-driven. While many patients appreciate the convenience and accessibility of virtual consultations, others prefer face-to-face care for reassurance or complex assessments [64]. Offering flexibility and preserving patient choice will ensure that telemedicine complements, rather than replaces, traditional care.

## Conclusions

Telemedicine has fundamentally transformed orthopaedic trauma follow-up care. What began as a pandemic-driven necessity has evolved into an essential component of modern practice. This evidence overwhelmingly supports continued integration. Patient satisfaction remains high, clinical outcomes are equivalent or superior to traditional care, and economic benefits are substantial. Providers have successfully adapted, with most reporting high satisfaction and increased efficiency. However, challenges persist, including technical barriers, digital literacy gaps, and concerns about physical examination limitations. Addressing these issues through education, appropriate patient selection, infrastructure investment, and supportive policies is essential. The future of orthopaedic trauma care lies in hybrid models that combine the convenience and accessibility of virtual consultations with the clinical rigor of in-person assessment. By maintaining flexibility and patient-centered approaches, telemedicine can enhance, rather than replace, traditional care delivery, ensuring equitable, sustainable, and high-quality outcomes for all patients.
